# The modification effect of temperature on the relationship between air pollutants and daily incidence of influenza in Ningbo, China

**DOI:** 10.1186/s12931-021-01744-6

**Published:** 2021-05-20

**Authors:** Rui Zhang, Yujie Meng, Hejia Song, Ran Niu, Yu Wang, Yonghong Li, Songwang Wang

**Affiliations:** 1 Chinese Center for Disease Control and Prevention, Beijing, 102206 China; 2 National Institute of Environmental Health, Chinese Center for Disease Control and Prevention, No 7. Panjiayuan Nanli, Chaoyang District, Beijing, 100021 China; 3 National Institute for Nutrition and Health, Chinese Center for Disease Control and Prevention, Beijing, 100050 China

**Keywords:** Influenza, Temperature, Air pollutants, Distributed lag non-linear model (DLNM), Ningbo

## Abstract

**Background:**

Although exposure to air pollution has been linked to many health issues, few studies have quantified the modification effect of temperature on the relationship between air pollutants and daily incidence of influenza in Ningbo, China.

**Methods:**

The data of daily incidence of influenza and the relevant meteorological data and air pollution data in Ningbo from 2014 to 2017 were retrieved. Low, medium and high temperature layers were stratified by the daily mean temperature with 25th and 75th percentiles. The potential modification effect of temperature on the relationship between air pollutants and daily incidence of influenza in Ningbo was investigated through analyzing the effects of air pollutants stratified by temperature stratum using distributed lag non-linear model (DLNM). Stratified analysis by sex and age were also conducted.

**Results:**

Overall, a 10 μg/m^3^ increment of O_3_, PM_2.5_, PM_10_ and NO_2_ could increase the incidence risk of influenza with the cumulative relative risk of 1.028 (95% CI 1.007, 1.050), 1.061 (95% CI 1.004, 1.122), 1.043 (95% CI 1.003, 1.085), and 1.118 (95% CI 1.028, 1.216), respectively. Male and aged 7–17 years were more sensitive to air pollutants. Through the temperature stratification analysis, we found that temperature could modify the impacts of air pollution on daily incidence of influenza with high temperature exacerbating the impact of air pollutants. At high temperature layer, male and the groups aged 0–6 years and 18–64 years were more sensitive to air pollution.

**Conclusion:**

Temperature modified the relationship between air pollution and daily incidence of influenza and high temperature would exacerbate the effects of air pollutants in Ningbo.

**Supplementary Information:**

The online version contains supplementary material available at 10.1186/s12931-021-01744-6.

## Introduction

Influenza is an acute respiratory infectious disease caused by influenza viruses which circulate in all parts of the world. It represents a year-round disease burden. The World Health Organization (WHO) reported that the global seasonal influenza episodes caused approximately 290–650 thousand deaths each year [1]. It causes illnesses that range in severity and sometimes lead to hospitalization and death. The most common symptoms are fever, cough, sore throat, runny nose, lack of appetite and fatigue. Most people recover from fever and other symptoms within a week without requiring medical attention. However, influenza can cause severe illness or death, particularly among high risk groups including the children younger than 6 years old, the elderly with chronic diseases, pregnant women and those with serious medical conditions [2].

An increasing number of studies have explored the risk factors of influenza. A few studies have demonstrated the relationship between weather and influenza in detail [2, 3]. Weather factors, especially temperature and humidity, play a significantly important role in the transmission of influenza. Temperature and humidity have been indicated to have an impact on host immune response and behavior changes [4, 5]. The extreme weather temperature can impact human health and the thermal stresses can lead not only to direct deaths and illnesses, but also to aggravation of respiratory diseases [6]. In temperate climates, seasonal epidemics occur mainly during winter, while in tropical regions, influenza may occur throughout the year, causing outbreaks more irregularly [1]. Therefore, the regional heterogeneity of influenza seasonality may be partly driven by complex effect of weather factors [7].

However, weather factors are not the only environmental risk factor associated with influenza. Many studies have proved that air pollution is also a significant environmental cause of influenza [7–10]. Air pollution has ever become a global major public health problem. Previous studies showed that air pollution has been associated with many adverse health problems, including respiratory disease [11, 12], cardiovascular diseases [13, 14], and death [15–18]. Studies have found that air pollution can not only make infected people sick by weakening the human immune system but also carry microorganisms to directly infect people [19]. Experiments have shown that air pollutants can affect airways through inhalation and affect the spread of influenza viruses. Exposure to O_3_ could result in sore throat, bronchial inflammation and pulmonary emphysema via stimulating respiratory tract [20]. Influenza virus can be attached to PM and suspended in the air, so that it can remain airborne long enough to be inhaled into the respiratory tract of a susceptible host [10]. Nitrogen dioxide may increase the susceptibility to respiratory viruses and the groups exposed to NO_2_ became more likely to be infected than those groups breathing clean air [21].

Global warming and air pollution pose a serious threat to health. Although these two factors will not work alone in reality, most studies have focused on the effect of climate change or air pollutions on health. In addition, some researches have studied the interaction between temperature and pollutants on various outcomes (such as emergency department visits [22], hospital admissions due to respiratory system diseases [12], acute mortality [16], and daily mortality [17–20]). However, to the best of our knowledge, temperature whether modified the relationship between air pollution and daily incidence of influenza remain poorly studied. Our study aims to assess the modification effect of temperature on the relationship between air pollutants and daily incidence of influenza in Ningbo. The results are expected to further understand the impacts of temperature and air pollution on incidence of influenza so as to provide scientific evidence for establishing more effective influenza early warning system.

## Materials and methods

### Study area

**Fig. 1:**
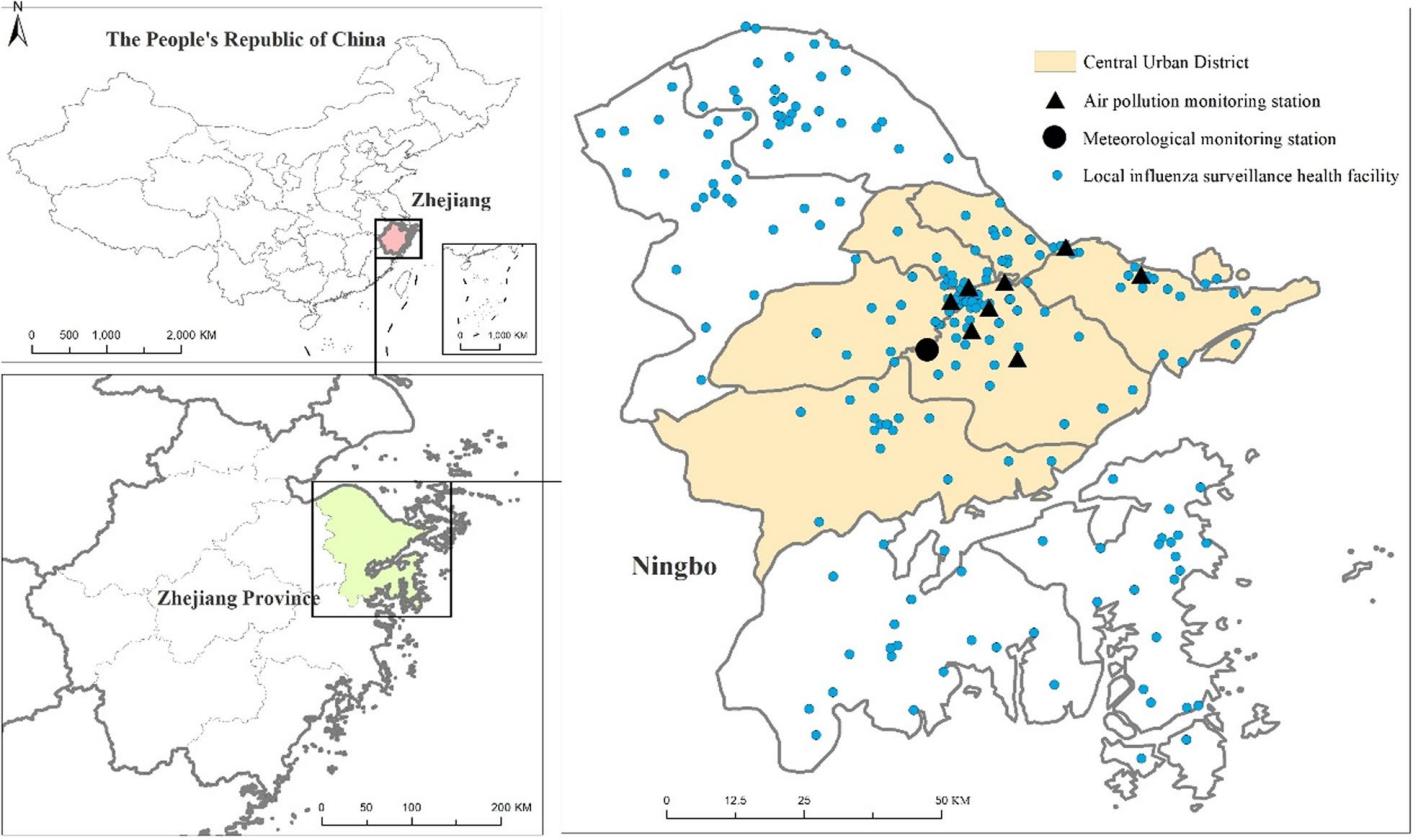
The spatial distribution of local influenza surveillance health facilities, meteorological monitoring station and air pollution monitoring stations in Ningbo city

Ningbo locates in the Southern China which composed of six districts, two counties and two county-level cities. Six districts are central urban districts, two counties and two county-level cities are suburbs (See Fig. 1). Its land area is 9714 km^2^, while oceanic territory amounts to 9758 km^2^. It is a port city with a residential population of 8.05 million at the end of 2017. It belongs to the north subtropical monsoon climate zone, which is mild and humid, with obvious alternation of winter and summer monsoon. It has four distinct seasons, with four months in winter and summer and two months in spring and autumn.

### Data collection

The daily incidence data of influenza in Ningbo from January 2014 to December 2017 were applied from the official website of The Data-center of China Public Health Science [23]. The daily incidence data were collected from 217 local influenza surveillance health facilities in Ningbo, including all hospitals at county level and above and all township hospitals (See Fig. 1). All the 217 local influenza surveillance health facilities are qualified to diagnose influenza. In the analysis, the following diagnosis codes of influenza, according to the International Classification of Diseases 10 (ICD-10), were used: J10.000, J10.001, J10.100, J10.101, J10.800, J10.801+ and J10.802+.

The daily mean temperature (℃), daily mean pressure (hPa) and daily mean relative humidity (%) were observed at Yinzhou station, the national meteorological monitoring station in the central city of Ningbo (See Fig. 1). These meteorological data were provided by the China Meteorological Administration.

The daily concentrations of each pollutant (daily maximum 8 h average ambient O_3_ concentration (O_3_; 8 h mean in μg/m^3^), daily average particular matter ≤ 2.5 μm (PM_2.5_; 24 h mean in μg/m^3^), daily average particular matter ≤ 10 μm (PM_10_; 24 h mean in μg/m^3^) and daily average nitrogen dioxide (NO_2_; 24 h mean in μg/m^3^)) were averaged from the available monitored results of eight stations which provided by Environmental Monitoring Center of Ningbo City (See Fig. 1).

### Methods

A distributed lag non-linear model (DLNM) with quasi-Poisson regression was applied to evaluate the association between air pollutant and incidence of influenza in Ningbo. The DLNM model was defined by the following formula:

$$\log \left[ {E\left( {Y_{t} } \right)} \right] = \alpha + cb\left( {X_{t,l} , df = 4} \right) + cb\left( {Tmean_{t,l} , df = 4} \right) + ns\left( {time_{i} , df = 7/year} \right) + ns\left( {RHmean, df = 3} \right) + \beta DOW_{t} + \gamma Holiday_{t}$$

where ${Y}_{t}$ represents the expected number of influenza incidence on day $t$. $\alpha$ is the intercept. $cb$ refers to the cross-basis function, which specifies the exposure-lag-response relationship simultaneously in the exposure–response and lag-response dimensions; $cb\left({X}_{t,l}\right)$ is a cross-basis function used to model the nonlinear lagged effects of daily independent pollutant variables; $X$ refers to the O_3_, PM_2.5_, PM_10_ and NO_2_, respectively; $Tmean$ is the daily mean temperature. $t$ means day $t$. *l* refers to the lag of the $X$/$Tmean$; The selection of degree of freedom was based on minimizing Akaike’s Information Criterion (AIC) [24]. A quadratic B spline (*bs*) with 4 degrees of freedom (*df*) was used for the exposure–response relationship and natural cubic splines (*ns*) with 4 degrees of freedom for the lag-response relationship which is consistent with the *df* ranges used in previous studies [25]. According to the Additional file 1: Fig. S1, 28, 7, 14, 14 and 14 were selected as the maximum lag days of *Tmean*, O_3_, PM_2.5_, PM_10_ and NO_2_, respectively [26]. *ns* is the natural cubic spline function in the R package ‘dlnm’ [27]. A smooth function of time with 7 degrees of freedom per year was used in the model to control the seasonality and long-term trends [28]. Daily mean relative humidity (*RHmean*), as potential confounding variables, was modeled as a natural cubic spline (*ns*) with 3 degrees of freedom. $DOW$ stands for day of the week, which was entered as a categorical variable, and $\beta$ is the coefficient of $DOW$ [27, 29–31].

Strong negative correlations were detected between Tmean and Pmean, with correlation coefficients of − 0.89 (P < 0.01) (Additional file 2: Table S1). To avoid multicollinearity, only Tmean and RHmean were considered as meteorological variables in the models.

The results of the analysis were expressed in cumulative relative risk (CRR) and their 95% confidence interval (CI) which was produced using regression coefficient for air pollutants in the above log-linear model. CRR means the overall effect of exposure on health outcomes over the possible lag periods. CRR was calculated for the interquartile range (75th percentile to 25th percentile) of the air pollution concentration for each pollutant, as observed during the study period [27, 28]. Furthermore, stratified analyses by temperature were conducted. In order to examine the interaction of weather temperature with the effects of air pollutants, the effects of each air pollutant were tested separately for different temperature levels. The temperature was divided into low, medium and high temperature layers by cut-points at the 25th and 75th percentiles of temperature (-4.5 ℃ to 10.1 ℃, 10.1 ℃ to 24.2 ℃, 24.2 ℃ to 32.9 ℃) [17]. In addition, we also estimated the effects by gender and age (0–6, 7–17, 18–64 and ≥ 65 years) separately. The statistical significance tests of pollutant effects between low temperature layer and medium/high temperature layer were conducted by using the formula below [32]:

$$\left( {\widehat{{Q_{1} }} - \widehat{{Q_{2} }}} \right) \pm 1.96\sqrt {S\widehat{{E_{1} }}^{2} + S\widehat{{E_{2} }}^{2} }$$

where $\widehat{{\mathrm{Q}}_{1}}\mathrm{ and }\widehat{{\mathrm{Q}}_{2}}$ are the estimates for the two categories, and $\mathrm{S}\widehat{{\mathrm{E}}_{1}}$ and $\mathrm{S}\widehat{{\mathrm{E}}_{2}}$ are their respective standard errors.

### Sensitivity analysis

The temperature was divided into low, medium and high temperature layers by cut-points at the 25th and 75th percentiles of temperature, respectively. Due to the inherent arbitrary selection of the cut-point values of temperature stratification analysis, a sensitivity analysis was carried out by changing the cut-point values (20th and 80th, 30th and 70th) to address the sensitivity of the estimated effects of air pollutants [17]. We also tested the stability of the model by changing the degree of freedom of air pollutants (3 and 5) and the lag days of Tmean (14), respectively. Then the statistical significance of estimated effects among the different temperature strata were tested.

The spatial distribution of local influenza surveillance health facilities, meteorological monitoring station and air pollution monitoring stations in Ningbo city were created using ArcGIS (Redlands, USA, version 10.6). Data analysis was conducted using R software 3.6.2 with the package ‘dlnm’ [27]. The ‘dlnm’ package was used for fitting DLNM. For all statistical tests, statistical significance as a two-tailed P < 0.05.

## Results

**Fig. 2:**
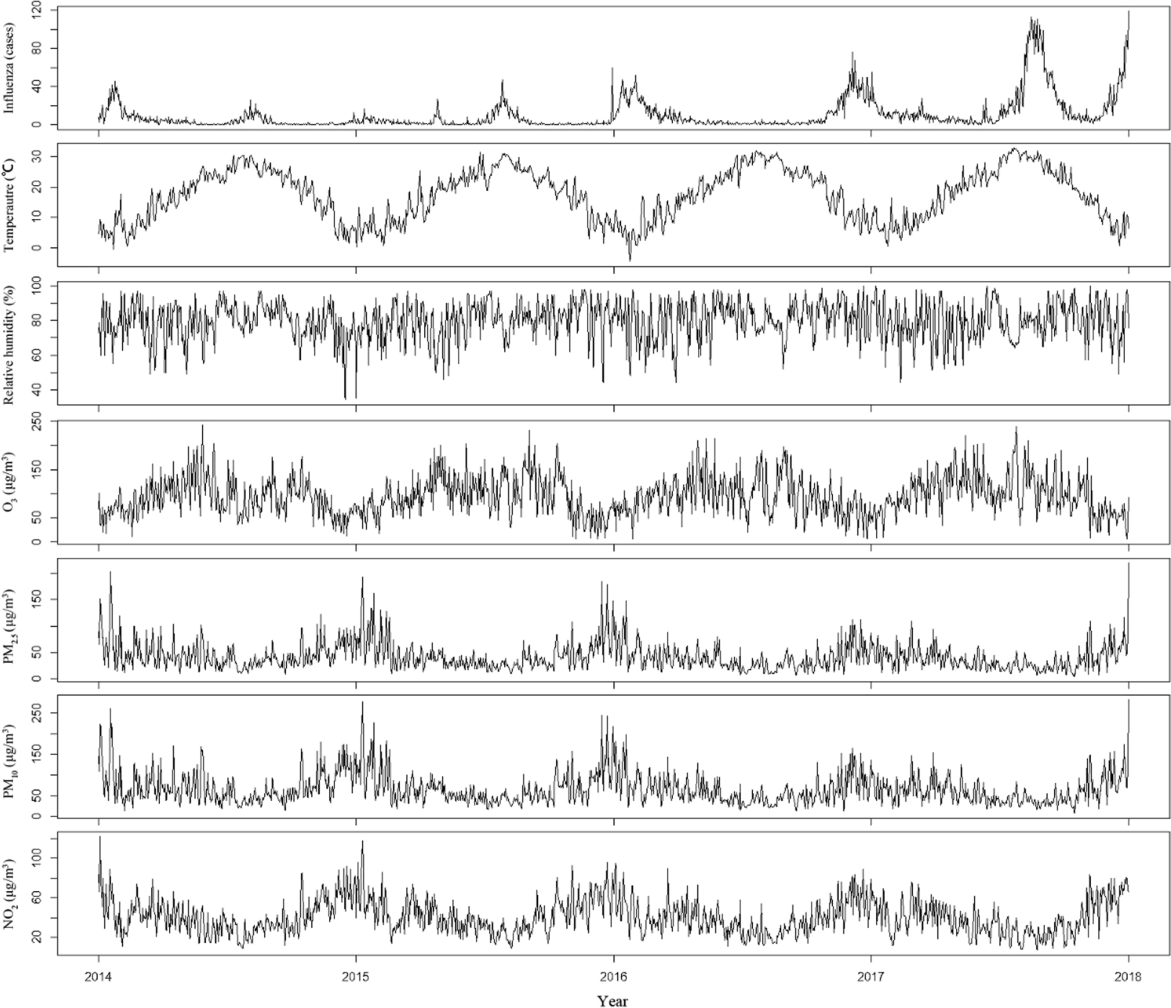
The time series distribution of daily incidence of influenza, meteorological variables and air pollutants in Ningbo, 2014–2017

There were 15,312 cases of influenza from January 1st, 2014 to December 31st, 2017, in Ningbo. Summary statistics for daily incidence of influenza cases, mean temperature and air pollutants were given in Table 1. There were approximately 10.48 influenza cases per day in the study area. Mean value of daily mean temperature was 17.47 °C. The mean values of O_3_, PM_2.5_, PM_10_ and NO_2_ were 95.52 μg/m^3^, 41.61 μg/m^3^, 65.94 μg/m^3^ and 40.39 μg/m^3^. Figure 2 shows the time-series distributions of daily incidence of influenza (Influenza), daily mean temperature (Tmean), daily mean pressure (Pmean), daily mean relative humidity (RHmean), O_3_, PM_2.5_, PM_10_ and NO_2_ during the study period. The daily incidence of influenza in Ningbo presented an upward long-term trend from 2014 to 2017. A bimodal seasonal pattern was observed, which was characterized by peaks in the summer (July) and winter (December). In addition, the daily mean concentrations of PM_2.5_, PM_10_, and NO_2_ were much lower in summer than winter and the trend of O_3_ was opposite.

**Table 1:** Descriptive statistics of daily incidence of influenza cases, meteorological factors and air pollutants in Ningbo, 2014–2017

Variables	Cases	Mean ± SD	Min	Percentile			Max
				25	50	75	
Influenza							
Total	15,312	10.48 ± 16.99	0	1	4	12	119
Gender							
Male	7445	5.10 ± 8.34	0	0	2	6	68
Female	7867	5.39 ± 8.99	0	0	2	6	62
Age							
0–6	4233	2.90 ± 4.73	0	0	1	4	32
7–17	3248	2.22 ± 5.36	0	0	0	2	50
18–64	6453	4.42 ± 8.60	0	0	2	5	66
≥ 65	1378	0.94 ± 2.18	0	0	0	1	22
Tmean (°C)	–	17.47 ± 8.41	− 4.5	10.1	18.5	24.2	32.9
Pmean (hPa)	–	1016.01 ± 8.83	985.7	1008.6	1015.7	1023.1	1039.7
Rhmean (%)	–	79.75 ± 11.18	34	73	81	88	100
O_3_ (μg/m^3^)	–	95.52 ± 40.77	6	66	91	121	242
PM_2.5_ (μg/m^3^)	–	41.61 ± 25.63	4	24	36	53	219
PM_10_ (μg/m^3^)	–	65.94 ± 37.16	7	39	57	82	282
NO_2_ (μg/m^3^)	–	40.39 ± 17.6	7	28	38	52	122

As Table 2 shown, overall, all air pollutants were significantly associated with daily incidence of influenza. A 10 μg/m^3^ increment of O_3_, PM_2.5_, PM_10_ and NO_2_ could increase the risk of daily incidence of influenza with the CRR of 1.028 (95% CI 1.007, 1.050), 1.061 (95% CI 1.004, 1.122), 1.043 (95% CI 1.003, 1.085), and 1.118 (95% CI 1.028, 1.216), respectively. The results displayed that male and the group aged 7–17 years were more sensitive to air pollutants. For male, the CRR for a 10 μg/m^3^ increment of O_3_, PM_2.5_, PM_10_ and NO_2_ was 1.038 (95% CI 1.012, 1.065), 1.099 (95% CI 1.027, 1.177), 1.071 (95% CI 1.022, 1.124) and 1.143 (95% CI 1.032, 1.266), respectively. The CRR of group aged 7–17 years was 1.074 (95% CI 1.008, 1.143), 1.064 (95% CI 0.925, 1.223), 1.062 (95% CI 0.966, 1.168) and 1.335 (95% CI 1.087, 1.639), respectively.

**Table 2:** Cumulative relative risk (CRR) and 95% confidence interval of stratified daily incidence of influenza associated with a 10 μg/m^3^ increase of air pollutants in different temperature layers in Ningbo from 2014 to 2017

		Overall	Low temperature	Medium temperature	High temperature
O_3_	Total	1.028 (1.007,1.050)*	0.936 (0.860,1.018)	1.029 (0.974,1.086)	1.047 (1.001,1.094)*
	Gender				
	Male	1.038 (1.012,1.065)*	0.975 (0.879,1.083)	1.048 (0.982,1.119)	1.048 (0.991,1.109)
	Female	1.019 (0.993,1.044)	0.900 (0.819,0.989)*	1.009 (0.943,1.079)	1.049 (0.991,1.110)
	Age				
	0–6	1.017 (0.983,1.051)	0.936 (0.815,1.074)	1.019 (0.934,1.111)	1.001 (0.929,1.079)
	7–17	1.074 (1.008,1.143)*	0.974 (0.832,1.140)	1.203 (1.051,1.378)*	1.008 (0.878,1.158)
	18–64	1.012 (0.988,1.036)	0.933 (0.842,1.034)	0.973 (0.907,1.043)	1.077 (1.017,1.141)*
	≥ 65	0.995 (0.954,1.039)	0.777 (0.622,0.971)*	0.915 (0.801,1.044)	1.072 (0.963,1.193)
PM_2.5_	Total	1.061 (1.004,1.122)*	0.946 (0.833,1.075)	1.109 (0.957,1.285)	1.518 (1.117,2.064)*
	Gender				
	Male	1.099 (1.027,1.177)*	0.952 (0.814,1.114)	1.098 (0.922,1.309)	2.120 (1.442,3.115)*
	Female	1.024 (0.959,1.094)	0.934 (0.810,1.078)	1.120 (0.935,1.342)	1.117 (0.750,1.666)
	Age				
	0–6	1.035 (0.945,1.134)	0.892 (0.733,1.086)	1.188 (0.952,1.482)	1.811 (1.067,3.073)*
	7–17	1.064 (0.925,1.223)	0.885 (0.681,1.149)	0.741 (0.467,1.176)	0.730 (0.279,1.910)
	18–64	1.017 (0.951,1.087)	0.980 (0.836,1.149)	1.184 (0.982,1.429)	1.564 (1.053,2.324)*
	≥ 65	0.982 (0.871,1.107)	0.828 (0.608,1.126)	0.802 (0.583,1.104)	1.564 (0.724.3.382)
PM_10_	Total	1.043 (1.003,1.085)*	0.956 (0.867,1.053)	1.089 (0.989,1.200)	1.338 (1.052,1.701)*
	Gender				
	Male	1.071 (1.022,1.124)*	0.954 (0.847,1.074)	1.110 (0.988,1.246)	1.786 (1.321,2.414)*
	Female	1.015 (0.969,1.064)	0.955 (0.856,1.066)	1.069 (0.950,1.204)	1.028 (0.754,1.402)
	Age				
	0–6	1.026 (0.963,1.093)	0.899 (0.771,1.047)	1.158 (1.000,1.432)*	1.610 (1.067,2.429)*
	7–17	1.062 (0.966,1.168)	0.901 (0.742,1.095)	0.924 (0.689,1.238)	0.908 (0.427,1.934)
	18–64	1.005 (0.958,1.055)	1.015 (0.898,1.148)	1.096 (0.967,1.241)	1.351 (0.994,1.837)
	≥ 65	0.986 (0.906,1.074)	0.854 (0.669,1.090)	0.853 (0.690,1.056)	1.255 (0.687,2.291)
NO_2_	Total	1.118 (1.028,1.216)*	0.850 (0.673,1.073)	1.434 (1.161,1.771)*	1.933 (1.230,3.038)*
	Gender				
	Male	1.143 (1.032,1.266)*	0.882 (0.658,1.183)	1.281 (0.988,1.659)	2.130 (1.206,3.764)*
	Female	1.093 (0.989,1.209)	0.817 (0.625,1.070)	1.611 (1.244,2.087)*	1.782 (0.981,3.236)
	Age				
	0–6	1.101 (0.961,1.261)	0.676 (0.464,0.987)*	1.404 (1.017,1.941)*	1.566 (0.742,3.307)
	7–17	1.335 (1.087,1.639)*	0.605 (0.357,1.025)	1.402 (0.747,2.628)	0.949 (0.206,4.375)
	18–64	1.065 (0.964,1.176)	1.221 (0.901,1.654)	1.522 (1.159,1.998)*	2.132 (1.160,3.918)*
	≥ 65	1.026 (0.863,1.220)	0.848 (0.456,1.575)	1.073 (0.650,1.771)	3.121 (0.969,10.052)

*P < 0.05

Compared with low temperature layer, the CRRs were higher at medium and high temperature layers. Especially at high temperature layer, male, the groups aged 0–6 years and 18–64 years posed higher associations with air pollutants. At high temperature layer, a 10 μg/m^3^ increment of O_3_, PM_2.5_, PM_10_ and NO_2_ could increase the risk of total daily incidence of influenza with the CRR of 1.047 (95% CI 1.001, 1.094), 1.518 (95% CI 1.117, 2.064), 1.338 (95% CI 1.052, 1.701) and 1.933 (95% CI 1.230, 3.038), respectively. And that of male was 1.048 (95% CI 0.991, 1.109), 2.120 (95% CI 1.442, 3.115), 1.786 (95% CI 1.321, 2.414) and 2.130 (95% CI 1.206, 3.764), respectively. The CRR of group aged 0–6 years was 1.001 (95% CI 0.929, 1.079), 1.811 (95% CI 1.067, 3.073), 1.610 (95% CI 1.067, 2.429) and 1.566 (95% CI 0.742, 3.307), respectively. The CRR of group aged 18–64 years was 1.077 (95% CI 1.017, 1.141), 1.564 (95% CI 1.053, 2.324), 1.351 (95% CI 0.994, 1.837) and 2.132 (95% CI 1.160, 3.918), respectively.

### Sensitivity analysis

The sensitivity analysis results were shown in Additional file 3: Table S2. By using (20th percentile to 80th percentile) and (30th percentile to 70th percentile) temperature stratification analysis method, as well as changing the degree of freedom of air pollutants and the lag day of Tmean, it was found that the research results were stable and reliable.

## Discussion

In our study, overall, we found that O_3_, PM_2.5_, PM_10_ and NO_2_ exposure significantly increased daily incidence of influenza in Ningbo. Exposure to high concentration of air pollution has been widely recognized as a threat to respiratory systems. Previous research conclusions have shown that elevated air pollutant concentrations were associated with increased influenza risk, which is consistent with our results. For example, the study conducted in Wuhan, China found that O_3_ and NO_2_ were associated with the risk of influenza [33]. Three studies indicated that PM exposure can exacerbate morbidity due to influenza [9, 34, 35]. A study conducted in Chongqing, China found that NO_2_ exposure could worsen the risk of influenza infection [36]. And Zhiwei Xu et al. reported that high concentrations of O_3_ and PM_10_ were associated with more pediatric influenza cases in Brisbane, Australia [8]. Nevertheless, there are some previous reports not in consistent with our study. The study conducted in Jinan did not observe that NO_2_ could increase the incidence of influenza [34]. The study conducted in Hefei showed that NO_2_ was negatively correlated with influenza but with no significant association [7]. This inconsistency may be due to different statistical methods, study regions, concentrations of air pollutants, sources of air pollutants, demographic characteristics and cultural background, etc. Therefore, further multi-city studies were needed in the future. There are several potential mechanisms for the associations between air pollution and the risk of influenza. As a highly reactive oxidant, O_3_ may decrease host defenses against bacterial and fungal infections in the airways and increase oxidative stress, which in turn affects the function of the respiratory system [37]. Exposure to high levels of PM pollution induces both airway epithelial damage and barrier dysfunction and results in a temporary immunosuppressive pulmonary microenvironment, which eventually may enhance morbidity from respiratory viral infection. Another mechanism is that exposure to air pollutants (such as PM and NO_2_) will reduce the capacity of macrophages to engulf the virus, and lead to a decrease of the inactivation of macrophage-dependent invasive pathogens, thus seriously aggravating inflammation.

Temperature and air pollution are often significantly correlated, and they sometimes interact to affect human health. Through the analysis stratified by temperature, we concluded that there was significant evidence that temperature modified the relationship between air pollution and daily incidence of influenza. In particular, high temperature enhanced the effect of pollutants on incidence of influenza. The result is similar to previous studies [16, 19, 33]. However, some other studies had the reverse results, which found that low temperature and high concentration of particulate matters might bring higher risk to influenza [8, 10]. The inconsistent results may be related to the various climate characteristics. Ningbo is a typical southern city in China and has high temperature and humidity in summer. Extreme heat and high relative humidity can exacerbate negative effects of air pollutants on health [19]. Although the underlying mechanism on interaction between temperature and air pollutants is unclear, previous studies have shown that extreme temperature might increase the workload of the respiratory system and induce adverse respiratory events [11, 12, 14, 22]. In addition, from biological perspectives, heat stress can disrupt the normal physiological thermoregulation, including increases in blood viscosity, plasma cholesterol level, and inflammatory responses [38, 39]. At high temperature, NO_2_ can cause inflammatory reactions in the respiratory system which are associated with increased respiratory death [21, 40–42]. All of these can increase immune system stress and alter a person’s physiologic response to toxic agents, so as to high temperature can increase the susceptibility to air pollutants.

In different population subgroups, we found that, overall, males and the group aged 7–17 years were more sensitive to air pollutants. While during high temperature days, males and groups aged 0–6 years and 18–64 years were more sensitive to air pollution. The difference in gender- and age-specific associations between air pollution and incidence of influenza might be attributable to the difference in exposure to the outdoor environments, such as home, schools, transportations, workplaces, etc. Males are more likely to do outdoor jobs than females, such as driving, transportation, construction and fishing. The high exposure to outdoor pollutants might partially explain why the effects of air pollution on males are almost consistently more pronounced compared with females. It was displayed in our study that, besides the group aged 18–64 years who are the mainstay of occupational population and have the high chance to expose to outdoor air pollutants, groups aged 0–6 years and 7–17 years also had high CRR values. It meant that preschool-age and school-age children be more susceptible to air pollutants, especially on hot days. Meanwhile, we found that the group aged 7–17 years were mainly affected by O_3_ and NO_2_ which are traffic/gaseous air pollutants, while the group aged 0–6 years seemed to be primarily impacted by PM_2.5_ and PM_10_ and not by O_3_ and NO_2_. It might because that school-aged children have more opportunities to expose to traffic pollution on the way to and from school. While preschool-age children spend more time indoors, but not outdoors and hence less exposed to traffic near-road pollutants such as O_3_ and NO_2_. Our results indicated that more attention should be paid to preschool-age and school-age children, and outdoor activities should be controlled to reduce their exposure to influenza viruses during the specific period of high air pollution especially with high ambient temperature. At the same time, hand hygiene and monitoring of symptoms and diseases in kindergartens and schools should be strengthened, especially in the flu season.

To the best of our knowledge, this is the first study to examine the modification effect of temperature on the relationship between air pollutants and daily incidence of influenza in Ningbo. Several weaknesses in this study should be acknowledged. First, this study employed an ecological study design and lacked personal exposure information. Second, the data of the study come from the Data-center of China Public Health Science which are based on the hospital reporting for the influenza monitoring cases. There may be selection or under-reporting bias, which may affect the precision of the association analysis. Third, there is only one meteorological monitoring station in Ningbo. It would cause exposure bias that the meteorological data measured by this only one station were used to represent the whole Ningbo area. Fourth, our study focused on Ningbo, a southern city in China. The findings need to be verified in more other cities or regions.

## Conclusion

The findings from our study enriched the scientific evidence of the modification effect of temperature on the relationship between air pollutants and daily incidence of influenza in Ningbo. The results can provide help to establish effective public health interventions and comprehensive warning systems that take into consideration both temperature and air pollution.

## Supplementary Information

**Additional file 1.Figure S1.** Relative risk of daily incidence of influenza associated with temperature on lag 0-30 days, O_3_ on lag 0-7 days, PM_2.5_ on lag 0-14 days, PM_10_ on lag 0-14 days and NO_2_ on lag 0-14 days.

**Additional file 2.Table S1.** Spearman correlation coefficients between Influenza, meteorological factors and air pollutants.

**Additional file 3.Table S2.** CRR of influenza incidence by changing the cut-points of temperature, degree freedom of air pollutants and lag of Tmean.

## Data Availability

All supporting data in this study can be applied from the website: http://www.phsciencedata.cn/Share/en/index.jsp.
