# Correction to: The modification effect of temperature on the relationship between air pollutants and daily incidence of influenza in Ningbo, China

**DOI:** 10.1186/s12931-021-01831-8

**Published:** 2021-09-07

**Authors:** Rui Zhang, Yujie Meng, Hejia Song, Ran Niu, Yu Wang, Yonghong Li, Songwang Wang

**Affiliations:** 1 Chinese Center for Disease Control and Prevention, Beijing, 102206 China; 2 National Institute of Environmental Health, Chinese Center for Disease Control and Prevention, No 7. Panjiayuan Nanli, Chaoyang District, Beijing, 100021 China; 3 National Institute for Nutrition and Health, Chinese Center for Disease Control and Prevention, Beijing, 100050 China

## Correction to: Respir Res (2021) 22:153 https://doi.org/10.1186/s12931-021-01744-6

The original version [1] of this article unfortunately contained a mistake. The presentation of Supplementary Fig. S1 was incorrect. The correct version is given (Additional file 1: Figure S1). The original article has also been corrected.

## Supplementary Information

**Additional file 1: Figure S1.** Relative risk of daily incidence of influenza associated with temperature on lag 0–30 days, O_3_ on lag 0–7 days, PM_2.5_ on lag 0–14 days, PM_10_ on lag 0–14 days and NO_2_ on lag 0–14 days.
