# Supplementary material for: The modification effect of temperature on the relationship between air pollutants and daily incidence of influenza in Ningbo, China
Source: Respir Res. 2021 May 20;22:153. doi: 10.1186/s12931-021-01744-6 (PMC8138986; doi:10.1186/s12931-021-01744-6)
Supplement: Supplementary file 1 — Additional file 1.Figure S1. Relative risk of daily incidence of influenza associated with temperature on lag 0-30 days, O3 on lag 0-7 days, PM2.5 on lag 0-14 days, PM10 on lag 0-14 days and NO2 on lag 0-14 days. [file 12931_2021_1744_MOESM1_ESM.docx]

**
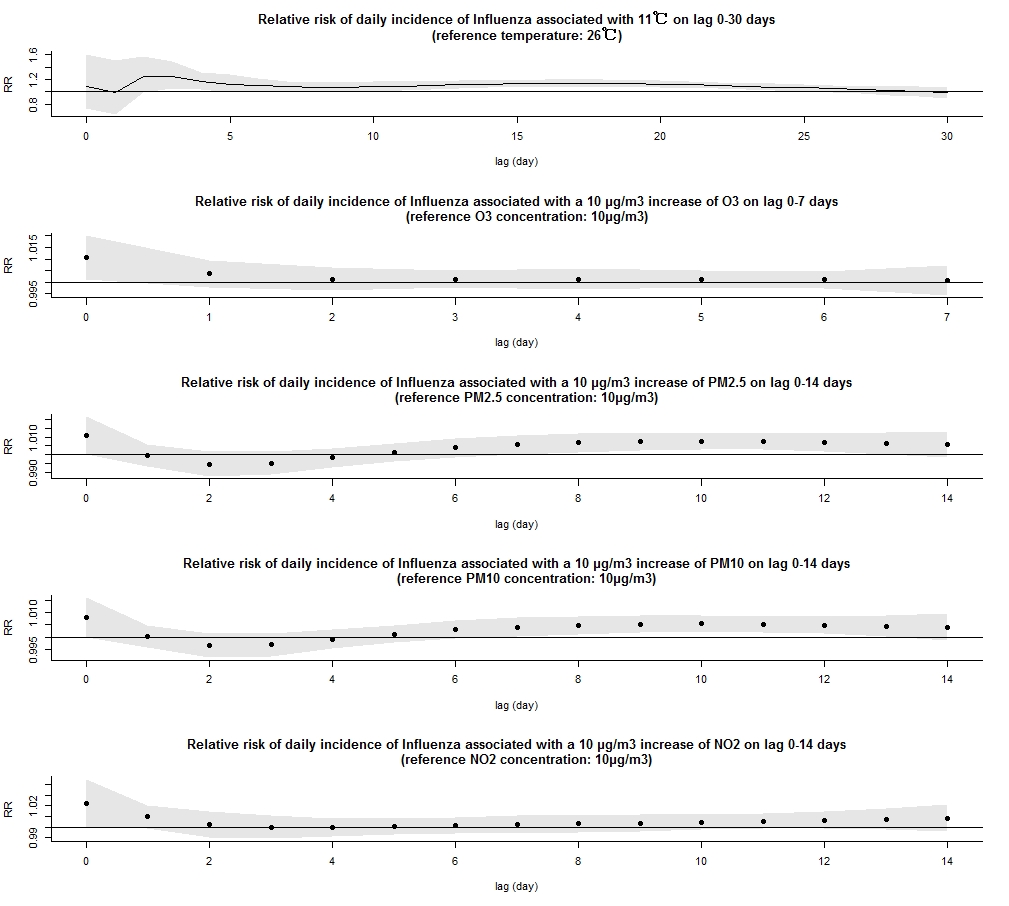
**

**Figure S1. Relative risk of daily incidence of influenza associated with temperature on lag 0-30 days, O_3_ on lag 0-7 days, PM_2.5_ on lag 0-14 days, PM_10_ on lag 0-14 days and NO_2_ on lag 0-14 days**
