# Supplementary material for: The modification effect of temperature on the relationship between air pollutants and daily incidence of influenza in Ningbo, China
Source: Respir Res. 2021 May 20;22:153. doi: 10.1186/s12931-021-01744-6 (PMC8138986; doi:10.1186/s12931-021-01744-6)
Supplement: Supplementary file 2 — Additional file 2.Table S1. Spearman correlation coefficients between Influenza, meteorological factors and air pollutants. [file 12931_2021_1744_MOESM2_ESM.docx]

**Table S1. Spearman correlation coefficients between Influenza, meteorological factors and air pollutants**

|  | **Tmean** | **Pmean** | **Rhmean** | **O_3_** | **PM_2.5_** | **PM_10_** | **NO_2_** |
| --- | --- | --- | --- | --- | --- | --- | --- |
| Influenza | -0.03 | 0.09** | -0.01 | -0.07** | 0.06* | 0.05 | 0.01 |
| Tmean |  | -0.89** | 0.15** | 0.39** | -0.48** | -0.49** | -0.57** |
| Pmean |  |  | -0.26** | -0.34** | 0.41** | 0.45** | 0.52** |
| RHmean |  |  |  | -0.36** | -0.08** | -0.18** | 0.09** |
| O_3_ |  |  |  |  | -0.04 | -0.02 | -0.25** |
| PM_2.5_ |  |  |  |  |  | 0.96** | 0.72** |
| PM_10_ |  |  |  |  |  |  | 0.75** |
| *: P < 0.05 | |  |  |  |  |  |  |
| **: P < 0.01 | |  |  |  |  |  |  |
