# Supplementary material for: The modification effect of temperature on the relationship between air pollutants and daily incidence of influenza in Ningbo, China
Source: Respir Res. 2021 May 20;22:153. doi: 10.1186/s12931-021-01744-6 (PMC8138986; doi:10.1186/s12931-021-01744-6)
Supplement: Supplementary file 3 — Additional file 3.Table S2. CRR of influenza incidence by changing the cut-points of temperature, degree freedom of air pollutants and lag of Tmean. [file 12931_2021_1744_MOESM3_ESM.docx]

**Table S2. CRR of influenza incidence by changing the cut-points of temperature, degree freedom of air pollutants and lag of Tmean**

|  |  | **Overall** | **Low temperature** | **Medium temperature** | **High temperature** |
| --- | --- | --- | --- | --- | --- |
| P20/P80 | O_3_ | 1.028(1.007,1.050)* | 0.955(0.833,1.095) | 1.090(1.046,1.136)* | 1.085(1.021,1.154)* |
|  | PM_2.5_ | 1.061(1.004,1.122)* | 0.891(0.732,1.085) | 1.233(1.119,1.358)* | 1.152(0.659,2.014) |
|  | PM_10_ | 1.043(1.003,1.085)* | 0.885(0.753,1.039) | 1.156(1.086,1.231)* | 1.042(0.711,1.526) |
|  | NO_2_ | 1.118(1.028,1.216)* | 0.682(0.426,1.092) | 1.646(1.385,1.957)* | 1.103(0.641,1.895) |
| P30/P70 | O_3_ | 1.028(1.007,1.050)* | 0.944(0.887,1.004) | 1.029(0.965,1.097) | 1.057(1.016,1.099)* |
|  | PM_2.5_ | 1.061(1.004,1.122)* | 0.873(0.773,0.987)* | 1.103(0.941,1.293) | 1.234(0.925,1.645) |
|  | PM_10_ | 1.043(1.003,1.085)* | 0.887(0.803,0.980)* | 1.113(1.003,1.234)* | 1.108(0.890,1.379) |
|  | NO_2_ | 1.118(1.028,1.216)* | 0.982(0.726,1.328) | 1.193(0.972,1.463) | 1.504(0.990,2.284) |
| df=3 | O_3_ | 1.053(1.029,1.078)* | 1.092(0.905,1.318) | 1.068(1.021,1.119)* | 1.131(1.019,1.254)* |
|  | PM_2.5_ | 1.048(1.005,1.093)* | 0.969(0.834,1.126) | 1.113(1.036,1.195)* | 1.893(1.232,2.910)* |
|  | PM_10_ | 1.033(1.003,1.064)* | 0.992(0.887,1.110) | 1.083(1.034,1.133)* | 1.586(1.152,2.183)* |
|  | NO_2_ | 1.066(1.001,1.136)* | 1.014(0.757,1.358) | 1.157(1.037,1.291)* | 2.261(1.323,3.863)* |
| df=5 | O_3_ | 1.022(1.003,1.040)* | 0.913(0.837,0.995)* | 1.038(0.983,1.097) | 1.061(1.012,1.112)* |
|  | PM_2.5_ | 1.062 (1.005,1.122)* | 0.878 (0.754,1.023) | 1.245 (1.043,1.485)* | 2.347 (1.516,3.632)* |
|  | PM_10_ | 1.049 (1.009,1.091)* | 0.916 (0.814,1.030) | 1.191 (1.063,1.335)* | 1.803 (1.281,2.536)* |
|  | NO_2_ | 1.115 (1.024,1.215)* | 0.778 (0.604,1.001) | 1.426 (1.150,1.768)* | 1.743 (1.105,2.750)* |
| Lag of Tmean=14 | O_3_ | 1.022(1.003,1.040)* | 0.914(0.844,0.991)* | 1.068(1.017,1.121)* | 1.085(1.031,1.140)* |
|  | PM_2.5_ | 1.059(1.002,1.120)* | 0.888(0.761,1.035) | 1.051(0.897,1.230) | 1.943(1.219,3.099)* |
|  | PM_10_ | 1.044(1.004,1.085)* | 0.947(0.851,1.054) | 1.060(0.957,1.175) | 1.691(1.157,2.470)* |
|  | NO_2_ | 1.079(0.990,1.177) | 0.820(0.646,1.041) | 1.176(0.951,1.454) | 1.917(1.179,3.116)* |
| *P<0.05; **CRR: cumulative relative risk; Tmean: daily mean temperature.** | | | | | |
